# Mullite-like SmMn_2_O_5_-Derived Composite Oxide-Supported Ni-Based Catalysts for Hydrogen Production by Auto-Thermal Reforming of Acetic Acid

**DOI:** 10.3390/ma17112490

**Published:** 2024-05-22

**Authors:** Hui Chen, Qi Chen, Xiaomin Hu, Chenyu Ding, Lihong Huang, Ning Wang

**Affiliations:** 1 State Key Laboratory of Geohazard Prevention and Geoenvironment Protection, Chengdu University of Technology, Chengdu 610059, China; 2 College of Materials and Chemistry & Chemical Engineering, Chengdu University of Technology, Chengdu 610059, China; 3 College of Environmental Science and Engineering, Beijing University of Technology, Beijing 100124, China

**Keywords:** acetic acid, auto-thermal reforming, hydrogen, mullite-derived solid solution

## Abstract

The x%Ni/Sm_2_O_3_-MnO (x = 0, 10, 15, 20) catalysts derived from SmMn_2_O_5_ mullite were prepared by solution combustion and impregnation method; auto-thermal reforming (ATR) of acetic acid (HAc) for hydrogen production was used to explore the metal-support effect induced by Ni loadings on the catalytic reforming activity and product distribution. The 15%Ni/Sm_2_O_3_-MnO catalyst exhibited optimal catalytic performance, which can be due to the appropriate Ni loading inducing a strong metal–support interaction to form a stable Ni/Sm_2_O_3_-MnO active center, while side reactions, such as methanation and ketonization, were well suppressed. According to characterizations, Sm_2_O_3_-MnO mixed oxides derived from SmMn_2_O_5_ mullite were formed with oxygen vacancies; nevertheless, loading of Ni metal further promoted the formation of oxygen vacancies, thus enhancing adsorption and activation of oxygen-containing intermediate species and resulting in higher reactivity with HAc conversion near 100% and hydrogen yield at 2.62 mol-H_2_/mol-HAc.

## 1. Introduction

Due to the increasing consumption of fossil fuels, there is an urgent need to explore sustainable energy alternatives in order to address the growing energy demand [1,2,3]. Hydrogen has emerged as a promising green energy carrier with a high energy content of 122 kJ/g, thus gaining considerable attention [4,5,6,7]. Currently, the predominant technique for hydrogen production is the thermochemical conversion of fossil resources; however, this approach unavoidably results in carbon emissions [8,9,10,11]. In contrast, biomass and its derivatives as raw materials for hydrogen production conform to both energy renewability and carbon neutralization [12,13]. Bio-oil, obtained through fast pyrolysis of biomass, can be fractionated into an oil phase and a water-rich phase. Acetic acid (HAc), as the major component of the water-rich phase with contents up to 35 wt.%, exhibits potential for hydrogen production via a catalytic reforming process.

Catalytic reforming of acetic acid includes steam reforming (SR, Equation (1)), catalytic partial oxidation (CPOX, Equation (2)) and auto-thermal reforming (ATR, Equation (3)). SR is endothermic, and a heating source is required to sustain the reaction, especially for the high-temperature steam reforming process, e.g., SR of methane, in which high energy consumption is needed to supply high-temperature steam. CPOX is an exothermic reaction; however, the introduction of excessive air can lead to oxidation of active metal in the catalyst; on the other hand, the lack of water in the raw materials results in a low hydrogen yield [14]. In comparison, ATR is a heat-balance reaction, which combines endothermic steam reforming and exothermic oxidative reforming. Due to the combination, the heat released by the partial oxidation can be used for steam reforming. By adjusting the ratio of acetic acid, H_2_O and O_2_ in the feed, the exothermic and endothermic reactions can be balanced, and a relatively higher hydrogen yield can be obtained [15].

CH_3_COOH + 2H_2_O → 2CO_2_ + 4H_2_            ΔH = 131.5 kJ/mol (1)

CH_3_COOH + O_2_ → 2CO_2_ + 2H_2_      ΔH = −350.6 kJ/mol (2)

25CH_3_COOH + 36H_2_O + 7O_2_ → 50CO_2_ + 86H_2_   ΔH = 0 (3)

Ni-based catalysts are commonly utilized for the ATR of HAc due to their high activity in breaking C-C and C-H bonds [16]. Previous investigations have revealed the intricate nature of the transformation pathway of HAc during the ATR process and proposed possible routes for the conversion of HAc over Ni-based catalysts, as listed in Equations (4)–(7) [17,18]. The HAc molecule adsorbing on the active sites usually undergoes dehydrogenation, deoxygenation and decarbonylation reactions (CH_3_COOH→CH_3_COO*→CH_3_CO*→CH_3_*), leading to the formation of CH_3_* intermediates.

It is noteworthy that the carbon-containing intermediates of CH_x_* can be converted into the byproducts of CH_3_COCH_3_ or C* species (Equations (9) and (10)), which are the major source of coke deposition on the catalyst surface.

CH_3_COOH → CH_3_COO* + H* (4)

CH_3_COO* → CH_3_CO* + O* (5)

CH_3_COO* → CH_3_* + COO* (6)

CH_3_CO* → CH_3_* + CO* (7)

CH_3_CO* + CH_3_* → CH_3_COCH_3_ (8)

2CO* → CO_2_ + C* (9)

CH_x_* → *x*H* + C* (10)

Considering the above issues, suitable carriers and additives are needed to stabilize active components and to achieve a high hydrogen yield by tuning the reaction pathway. It has been reported that manganese oxides exhibit multivalent properties, including Mn^2+^, Mn^3+^ and Mn^4+^ ionic states [19,20]. The redox reactions occurring between these multivalent states can generate surface oxygen species and lattice oxygen vacancies, which in turn enhances the mobility of oxygen species and facilitates gasification of carbon precursors (C*) [21,22,23,24,25]. For example, Deng et al. reported that the incorporation of Ag through a hydrothermal method promoted the formation of oxygen defects within the MnO*_x_* matrix, while the abundant reactive oxygen species facilitated the combustion of volatile organic compounds (VOCs) and the elimination of O_3_ [26]. However, poor thermal stability of MnO or Mn_2_O_3_ still resulted in sintering at high temperatures [27]. The Sm_2_O_3_ species, as a basic material with thermal stability, can be used as a composite oxide carrier to improve resistance to sintering and to reduce the acidity of manganese oxides [28]. In addition, Sm_2_O_3_ as an oxidant is beneficial for gasification of coking precursors during the reforming process; for example, it was found that due to the presence of Sm, catalysts of Sm_2_O_3_-CeO_2_/Al_2_O_3_ and Rh-Sm_2_O_3_/CeO_2_-Al_2_O_3_ generated more oxygen vacancies, which promoted the catalytic cycle for carbon gasification [29].

Mullite-like SmMn_2_O_5_ possesses a special orthogonal crystal structure, in which the Mn element in the lattice presents different valence states and coordination environments, generating non-stoichiometric oxygen species and increasing surface oxygen vacancies. Zheng et al. [30] studied the catalytic oxidation mechanism of Mn-based mullite-type oxides and found that unlike traditional sequential reactions, there was a synergistic effect between adjacent lattice oxygen species, which resolved the contradiction between lattice oxygen stability and oxidation activity and exhibited high catalytic activity and stability with high-temperature conditions.

The metal loading also affects the selectivity and stability of nickel-based catalysts. It has been reported that Ni loading plays a crucial role in the reaction pathway of methane and ethanol steam reforming and has resulted in variation in hydrogen selectivity and the formation of coke; e.g., over the x%Ni/Ce_0.74_Zr_0.26_O_2_ (x = 10, 20, 30, 40) catalysts, ethanol conversion became higher with an increase in Ni loading, while the variation of conversion with Ni loading was related to the reducibility of the Ni species [31,32]. Generally, the Ni loading on different supports presents an optimal value, which induces appropriate interaction and stabilizes the active center, resulting in maximum hydrogen production and minimum carbon deposition in the reforming process [31,32]. 

Based on the above, we used the SmMn_2_O_5_ mullite structure as a precursor to load different contents of NiO and modified the structure by valence variation of the Mn species during the reduction process to regulate the metal–support interaction. The effect of Ni loading on the metal–support interaction and the conversion pathway in ATR of acetic acid was explored via different characterization methods. 

## 2. Experimental

### 2.1. Catalyst Preparation

The SmMn_2_O_5_ support was synthesized by the solution combustion method. Mn(CH_3_COO)_2_·4H_2_O (AR, Damao Chemicals, Tianjin, China) and Sm(NO_3_)_3_·6H_2_O (AR, Aladdin, Shanghai, China) with set ratios as listed in Table 1 were dissolved in the mixed solution of ethylene glycol/methanol with volume ratio at 3/2. The obtained mixture was stirred at room temperature for 24 h, dried in an oven at 80 °C for 8 h, transferred into a muffle furnace at 500 °C for 8 h and calcined in a tubular furnace at 800 °C for 8 h. 

The impregnation method was employed to load the Ni species. The SmMn_2_O_5_ support was added into the solution of Ni(NO_3_)_2_·6H_2_O (AR, Kelong Chemicals, Liaoning, China) and stirred at 60 °C until the solution was completely evaporated. Then, the obtained solid was dried at 80 °C for 16 h and calcined in air at 750 °C for 5 h. As shown in Table 1, the obtained x%Ni/Sm_2_O_3_-MnO catalysts (x = 0, 10, 15, 20) with different loadings of NiO were denoted as SM, 10NSM, 15NSM and 20NSM, respectively.

### 2.2. Catalytic Performance Test

ATR of HAc was conducted in a fixed-bed quartz tubing reactor with an inner diameter of 4.0 mm. Before the reaction, 0.1 g of catalyst (20–40 mesh) diluted with 0.3 g of quartz sand (20–40 mesh) was loaded into the middle of the quartz tube and reduced in hydrogen (100%, 30 mL·min^−1^) at 700 °C for one hour. An aqueous solution of HAc was pumped into an evaporator (230 °C) by a liquid pump (Elite, Dalian, China) and mixed with O_2_ and N_2_ with a molar ratio of HAc:H_2_O:O_2_:N_2_ at 1:4:0.28:3, while N_2_ was used as an internal standard for calibration. The feeding gas was introduced into the reactor with gas hour space velocity (GHSV) at 51,000 mL g_catal_^−1^ h^−1^ if not specified. Gas chromatography (SC-3000B, Chuanyi, Chongqing, China) equipped with a thermal conductivity detector (TCD) and flame ionization detector (FID) was used for online analysis of products. The HAc conversion (X_HAc_), selectivity of carbon-containing products (S_i_), hydrogen yield ($Y_{H_{2}}$), HAc conversion ($R_{\mathrm{HAc}}$), dispersion of Ni on the catalyst surface (D_Ni_) and turnover frequency (TOF) were calculated by Equations (11)–(16), respectively.

(11) $$X_{\mathrm{HAc}}=\frac{F_{\mathrm{HAc}\;\mathrm{in}}-F_{\mathrm{HAc}\;\mathrm{out}}}{F_{\mathrm{HAc}\;\mathrm{in}}}$$

(12) $$S_{i\;\mathrm{product}\;}=\frac{F_{i}}{n_{i}(F_{\mathrm{HAc}\;\mathrm{in}}-F_{\mathrm{HAc}\;\mathrm{out}})}$$

(13) $$R_{\mathrm{HAc}}=\frac{F_{\mathrm{HAc}\;\mathrm{in}}-F_{\mathrm{HAc}\;\mathrm{out}}}{g_{\mathrm{cat}}}$$

(14) $$R_{\mathrm{HAc}}=\frac{F_{\mathrm{HAc}\;\mathrm{in}}-F_{\mathrm{HAc}\;\mathrm{out}}}{g_{\mathrm{cat}}}$$

(15) $$D_{\mathrm{Ni}}=\frac{n_{{\mathrm{Ni}}_{\mathrm{surf}}^{0}}}{n_{\mathrm{Ni}}}$$

(16) $$\mathrm{TOF}=\frac{R_{\mathrm{HAc}}}{n_{{\mathrm{Ni}}_{\mathrm{surf}}^{0}}}$$

In the above equations, F_i, in or out_ represents the molar flow of i species at the inlet or outlet of the reactor; n_i_ is the ratio of carbon between carbon-containing products and HAc, while $g_{\mathrm{cat}}$ denotes the mass of catalysts. $n_{{\mathrm{Ni}}_{\mathrm{surf}}^{0}}$ signifies the mole of surface Ni^0^ per gram of catalyst. 

### 2.3. Catalyst Characterizations

X-ray diffraction (XRD) was investigated via Rigaku Ultima IV X-ray diffractometer (Tokyo, Japan) with Cu-Kα radiation at 40.0 kV and 30 mA from 5° to 80°. 

Nitrogen physisorption was carried out at −196 °C on a JW-BK112 automatic adsorption instrument (JWGB, Beijing, China). The specific surface area was calculated by Bruner–Emmett–Teller (BET) equation, and the pore-size distribution and pore volumes were calculated by the Barrett–Joyner–Halenda (BJH) model.

X-ray photoelectron spectroscopy (XPS) was recorded by an Ultra DLD spectrometer (Kratos, Manchester, UK) using Al Kα radiation (1486.8 eV), and the instrument accuracy is ±0.05 eV. The binding energy of adventitious carbon was settled at 284.6 eV to correct the charge effects. 

Temperature-programmed reduction (H_2_-TPR) was carried out in 5%H_2_/95%N_2_ gas from 50 °C to 900 °C on a TP-5076 apparatus (Xianquan Instrument, Tianjin, China) to evaluate the reducibility of the catalysts. 

A SC-200G apparatus (Chuanyi Instrument, Chongqing, China) fitted with TCD was used to record the temperature-programmed desorption of H_2_ (H_2_-TPD). A total of 200 mg of catalyst powder was pretreated in a flow of 5%H_2_/95%N_2_ at 700 °C for 30 min, then cooled down and maintained at 50 °C for 30 min. After purging with N_2_ for 30 min, the desorption of hydrogen was performed in the N_2_ flow with the temperature increasing from 50 °C to 700 °C at 10 °C·min^−1^. The amount of hydrogen desorption during the heating process was recorded by TCD.

To probe the coke formation during the reaction, thermogravimetry (TG) and differential thermal analysis (DTA) of spent catalysts were conducted on a SHIMADZU DTG-60 apparatus (Kyoto, Japan) in air by heating up to 900 °C with a heating ramp of 10 °C·min^−1^.

The morphologic features of the catalysts were characterized by scanning electron microscopy (SEM, Inspect F50, FEI, Hillsboro, OR, USA). The catalyst powder was sputtered with gold in a vacuum chamber prior to measurement.

## 3. Results and Discussion

### 3.1. Characterization of Catalyst Oxides

**Figure 1:**
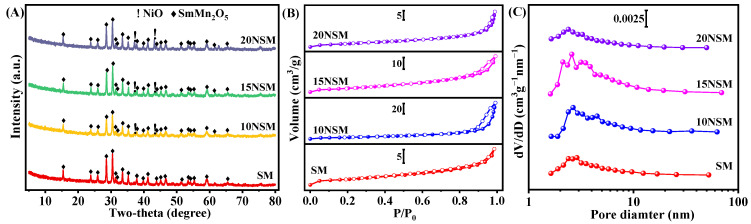
(**A**) XRD patterns, (**B**) N_2_ adsorption–desorption isotherms (solid symbols: adsorption; hollow symbols: desorption) and (**C**) pore-size distribution curves of calcined xNSM catalysts.

The catalysts calcined at 800 °C were characterized by XRD, as shown in Figure 1A. For the SM support, strong diffraction peaks of SmMn_2_O_5_ (PDF#97-008-4671) were observed, indicating that the mullite-like precursor of SmMn_2_O_5_ was successfully fabricated. After impregnating the Ni component, the peaks of the NiO (PDF#04-006-6160) phase appeared and intensified with the increasing content of Ni.

The specific surface area, pore volume and pore size of xNSM catalysts were measured by nitrogen physisorption, as presented in Figure 1B,C. According to the IUPAC classification, type IV isotherms with hysteresis loops were observed on all catalysts [33]. The hysteresis loop of SM was assigned to the H4 type, indicating that there were mainly slit-shaped pores [33,34], while the hysteresis loops of 10NSM, 15NSM and 20NSM were attributed to the H3 type, suggesting that the three catalysts mainly existed wedge-shaped pores, which are usually observed in aggregates of plate-like particles [33]. As shown in Table 1, the specific surface area first increased and then decreased with the increase in Ni. The 15NSM catalyst presented a higher specific surface area of 15.1 m^2^/g with pore volume and pore size at 0.048 cm^3^/g and 2.6 nm, respectively. The smallest specific surface area was found in the 20NSM catalyst, which could be attributed to the aggregation of excess Ni species on the support, resulting in the blockage of pore channels and a decrease in specific surface area [35]. 

### 3.2. Characterization of Reduced Catalysts

**Figure 2:**
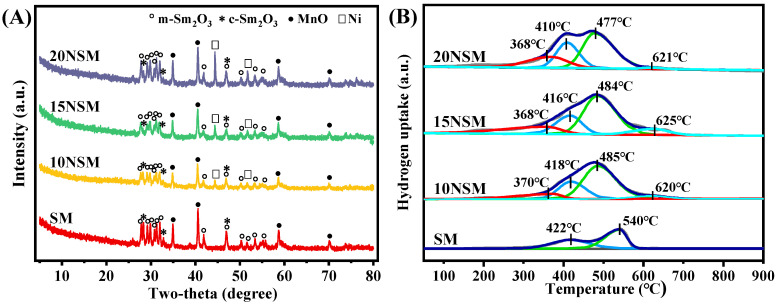
(**A**) XRD patterns of reduced xNSM catalysts and (**B**) H_2_-TPR profiles of xNSM catalysts.

To investigate the crystal structure of the xNSM catalysts after hydrogen reduction at 700 °C, XRD characterization was performed, as shown in Figure 2A. For the SM catalyst without Ni, the mullite-like SmMn_2_O_5_ precursor disappeared, while mixed phases of monoclinic Sm_2_O_3_ (m-Sm_2_O_3_, PDF#97-003-4291) and cubic Sm_2_O_3_ (c-Sm_2_O_3_, PDF#97-002-7741) were observed. The peaks of MnO (PDF#04-003-7163) were also found, suggesting that Mn^3+^/Mn^4+^ species in SmMn_2_O_5_ were reduced to Mn^2+^. Similar peaks of m-Sm_2_O_3_, c-Sm_2_O_3_ and MnO were found over all the Ni-containing catalysts, along with the peaks of Ni^0^ (PDF#97-005-3807). In addition, particle sizes of Ni^0^ were calculated by the Scherrer formula (d = k λ/(β cos θ)), as shown in Table 1. It can be seen that with the increase in metal loading, the Ni^0^ particle sizes gradually increased.

H_2_-TPR was performed to further investigate the reducibility of xNSM catalysts, as displayed in Figure 2B. For the SM catalyst, two reduction peaks near 422 °C and 540 °C can be attributed to the reduction of Mn^4+^ (Mn^4+^→Mn^3+^) and Mn^3+^ (Mn^3+^→Mn^2+^), respectively [36]. Over all the Ni-containing catalysts, four reduction peaks could be found. The peak near 370 °C can be attributed to the reduction of surface or amorphous NiO species, while the second and third peak can be assigned to the two-step reduction of Mn species. As compared with those of the SM catalyst, the reduction peaks of the Mn species gradually intensified, which was caused by the overlap of the reduction peak of NiO that weakly interacted with the support [37]. Moreover, the reduction peaks of the Mn species shifted to a lower temperature, indicating that Ni can promote the reduction of Mn species. The weak peaks near 620 °C can be ascribed to the NiO species that strongly interacted with the support [38,39]. 

**Figure 3:**
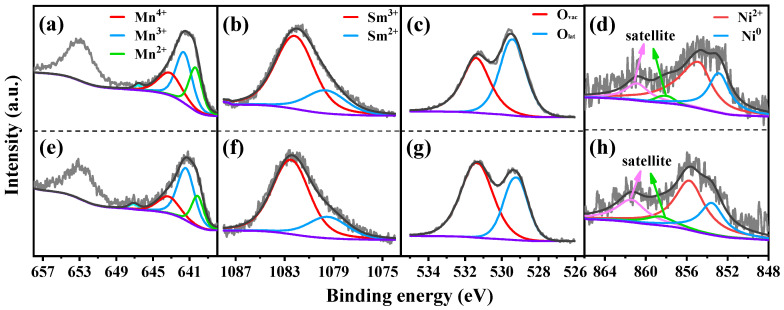
Mn 2p, Sm 3d and O 1s XPS spectra of reduced SM (**a**–**c**) and 15NSM (**e**–**g**) catalysts; Ni 2p_3/2_ XPS spectra of (**d**) reduced and (**h**) spent 15NSM catalyst (dark gray: fit curve; light gray: raw data; purple: background).

XPS was conducted to explore the valence state of elements in reduced catalysts. For the SM catalyst shown in Figure 3a, three peaks in the Mn 2p spectra near 640.5 eV, 641.7 eV and 643.3 eV can be assigned to Mn^2+^, Mn^3+^ and Mn^4+^, respectively [40,41]. The weak peak around 646.8 eV was attributed to the satellite peak of MnO, which is used to distinguish between MnO and other Mn oxides [42,43]. As compared to the SM catalyst, the binding energies of three peaks (640.2 eV, 641.5 eV, 643.2 eV) in the Mn 2p region of the 15NSM catalyst all migrated towards lower binding energies, which was the result of electron transfer between Sm and Mn elements, like Mn^3+^ + Sm^3+^ ↔ Mn^4+^ + Sm^2+^. Then, according to quantitative calculation (Table 2), the ratio of Mn^3+^/(Mn^2+^ + Mn^3+^ + Mn^4+^) over SM was 43%. It was owing to Sm species, which can lead to an appropriate number of structural defects, resulting in a considerable increase in Mn^3+^ species [44]. Over the 15NSM catalyst (Figure 3e), the ratio of Mn^3+^/(Mn^2+^ + Mn^3+^ + Mn^4+^) was recorded at 55%, which was higher than that of SM (43%), suggesting that because of the appropriate addition of Ni, a redox reaction was promoted. It also showed that more Mn^3+^ was exposed over the surface of 15NSM catalyst. The content of Mn^3+^ is crucial for the formation of oxygen vacancies, as Mn^3+^ can simultaneously transform into Mn^2+^ and Mn^4+^ [45,46]. Moreover, the Mn^3+^ species also can provide electrons that migrate toward Ni^2+^ and thus enhance the interaction between Ni and the support [47,48].

The XPS spectra of Sm 3d are shown in Figure 3b,f. Over the SM catalyst, the peaks near 1082.1 eV and 1079.6 eV were assigned to the binding energy of Sm^3+^ and Sm^2+^, respectively. The Sm^2+^/(Sm^2+^ + Sm^3+^) ratio at 36% over 15NSM was higher than that of the SM catalyst (29%), indicating that Sm^3+^ in 15NSM catalyst enriched more electrons from Mn^3+^ and was converted into Sm^2+^, which can generate more oxygen vacancies to promote the gasification of C* [44].

The XPS of O 1s spectra were deconvoluted into two peaks, as shown in Figure 3c,g. For the SM catalyst, the peak near 529.2 eV was ascribed to lattice oxygen (O_lat_) bonding to metal cations, and the peak around 531.3 eV can be corresponded to the defect sites with low oxygen coordination (oxygen vacancies, O_vac_) [49,50]. The relative content of O_vac_ reached 42% (Table 2), which is consistent with the above analysis that electron transfer promoted the generation of oxygen vacancies. For the 15NSM catalyst loaded with Ni, similar oxygen species were found; however, 15NSM showed a higher O_vac_ content (64%) than that of bare SM (42%), and this can be attributed to interaction between Ni and the support that further induced generation of oxygen vacancies [36]. The results indicate that more oxygen vacancies were formed over the 15NSM catalyst and that they were beneficial to promote the migration of oxygen species and activation of acetic acid [51].

For the Ni species in the reduced 15NSM catalyst in Figure 3d, the binding energy observed at 855.1 eV and 852.6 eV can be assigned to Ni^2+^ and Ni^0^, while the satellite peaks of Ni^2+^ and Ni^0^ were detected near 860.6 eV and 858.2 eV, respectively. In addition, the quantitative analysis of Ni^0^/(Ni^0^ + Ni^2+^) ratio was recorded at 36%, as listed in Table 2.

### 3.3. Catalytic Performance of the xNSM Catalyst

#### 3.3.1. Reactivity in ATR of HAc

**Figure 4:**
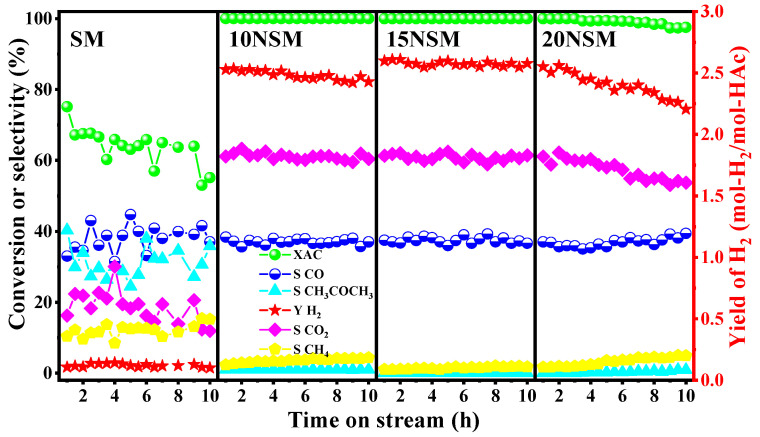
Catalytic performance of xNSM catalysts at 700 °C, GHSV at 51,000 mL·g_catal_^−1^·h^−1^ for ATR of HAc.

To investigate the catalytic performance, a series of xNSM catalysts were tested in the ATR of HAc for 10 h at 700 °C with GHSV at 51,000 mL·g_catal_^−1^·h^−1^, as shown in Figure 4. For the SM catalyst without Ni, acetic acid was not completely converted during the ATR process, and the HAc conversion gradually decreased from 75.2% to 55.2%; almost no hydrogen was detected. The selectivity to CO_2_ and CO was about 16.3% and 39.4%, respectively. Meanwhile, the selectivity to by-products of CH_4_ and CH_3_COCH_3_ was up to 12.1% and 31.1%, respectively. The results indicate that the SM catalyst presented poor activity in the absence of active Ni.

For catalysts loaded with Ni metal, the reaction activity was significantly improved, which was attributed to the remarkable activity of the Ni metal for breaking C-C and C-H bonds. Over the 10NSM catalyst, the conversion of acetic acid remained stable at 100% with an initial hydrogen yield of 2.57 mol-H_2_/mol-HAc. The selectivity to CO_2_ and CO was around 60.7% and 36.5%, respectively. However, the hydrogen yield showed a decreasing trend over time, while methane gradually increased. After 10 h of reaction, the hydrogen yield dropped to 2.42 mol-H_2_/mol-HAc, and the selectivity to methane increased to 3.8%.

The 15NSM catalyst exhibited relatively stable catalytic performance and activity during the reaction: acetic acid conversion remained at 100%, and hydrogen yield was stable at 2.62 mol-H_2_/mol-HAc. The selectivity to CO_2_ and CO was around 61.1% and 37.3%, respectively. Meanwhile, almost no by-products, such as methane and acetone, were detected during the reaction, indicating that the methanation and ketonization were well suppressed over the 15NSM catalyst.

For 20NSM loaded with 20% NiO, the initial conversion of acetic acid reached 100%, and the initial hydrogen yield also reached 2.52 mol-H_2_/mol-HAc. Nevertheless, with the reaction progressing, the conversion of acetic acid gradually decreased to 97.0% with the hydrogen yield decreasing to 2.19 mol-H_2_/mol-HAc, while the selectivity to the by-product of CH_4_ increased from 1.75% to 4.9%.

In conclusion, the catalytic activity of SM support was poor, and there was almost zero selectivity for hydrogen. After loading with active metal Ni, the catalytic activity was greatly improved, which was attributed to the formation of Ni/Sm_2_O_3_-MnO active centers that could effectively adsorb and activate the acetic acid molecules; meanwhile, side reactions, such as methanation and ketonization, were suppressed by the synergistic effect of Sm_2_O_3_ and MnO supports. When Ni loading was too high, the catalytic performance was reduced, which could be attributed to migration and the aggregation of larger Ni particles as formed. Therefore, the 15NSM catalyst exhibited the best catalytic performance and stability.

#### 3.3.2. The Turnover Frequency and Apparent Activation Energy

**Figure 5:**
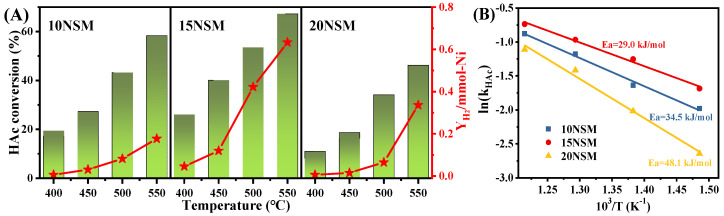
(**A**) The HAc conversion at 400–500 °C with GHSV at 338,000 mL·g_catal_^−1^·h^−1^ in ATR and hydrogen yield over per millimole of Ni atom (red star: Y_H2_/mmol-Ni); (**B**) apparent activation energy over the xNSM.

To further explore the reactivity of xNSM catalysts, the turnover frequency (TOF) and apparent activation energy (Ea) for ATR were calculated, while the dispersion of surface nickel species was calculated from H_2_-TPD by assuming ${n_{H}/n}_{{\mathrm{Ni}}_{\mathrm{surf}}^{0}\;}=$ 1. As shown in Figure 5A, the HAc conversions over the xNSM catalysts were tested at lower temperatures (400–550 °C) with a high GHSV at 338,000 mL·g_catal_^−1^·h^−1^ to better evaluate the kinetics and to avoid the influence of diffusion [52]. It can be found that even at low temperatures, the conversion of acetic acid over the 15NSM catalyst was still higher than those of 10NSM and 20NSM; moreover, the hydrogen yield per mmol surface Ni atoms using the 15NSM catalyst was significantly higher than those of 10NSM and 20NSM, indicating that the 15NSM catalyst enhanced the conversion of acetic acid for hydrogen. At 400 °C, HAc conversions over all catalysts were below 30%, which ensured that the obtained kinetic data were reliable [1,53]. Based on the dispersion and acetic acid conversion, TOF was calculated by Equation (13) and is shown in Table 3. The TOF of the 15NSM catalyst was recorded at 0.78 × 10^−2^·s^−1^, which is higher than those of the 10NSM catalysts, indicating that the Ni^0^ species in the 15NSM catalyst were more active to convert HAc. With increasing Ni content, the catalysts also showed lower TOF, with 0.62 × 10^−2^·s^−1^ in the 20NSM catalysts, which was owing to excessive Ni metallic agglomeration, further leading to an increase in particle size, which affects the dispersion of active molecules on the catalyst surface [54]. As reported by Ali Nakhaei et al. [55], the work exhibited a trend of increasing TOF with decreasing Ni-Mg particle size for the reforming of methane. The Ea of catalysts was calculated by the Arrhenius equation ($k={\mathrm{Ae}}^{-\mathrm{Ea}/\mathrm{RT}}$), as shown in Figure 5B. The 15NSM catalyst presented a lower Ea value of 29.0 kJ/mol, confirming the higher reactivity for HAc conversion.

#### 3.3.3. Catalytic Performance of Catalysts on O_2_/HAc and Different Temperatures

**Figure 6:**
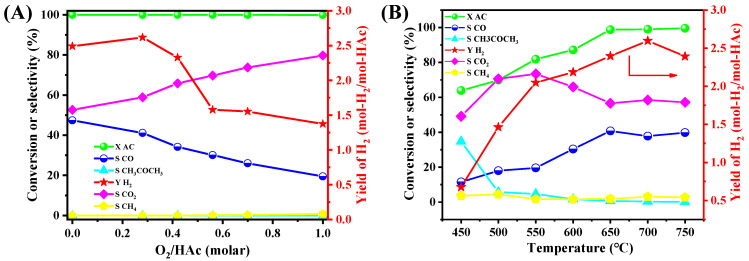
Effect of (**A**) O_2_/HAc molar ratios and (**B**) reaction temperatures on the catalytic performance of 15NSM catalyst in ATR of HAc (with GHSV at 51,000 mL·g_catal_^−1^·h^−1^) (red arrow: point to Y-axis).

The 15NSM catalyst exhibited higher catalytic performance during the ATR test and was then selected to investigate the effect of various reaction conditions. For the effect of O_2_/HAc in Figure 6A, the 15NSM catalyst presented a hydrogen yield of 2.49 mol-H_2_/mol-HAc with O_2_/HAc = 0, and only a trace of CH_4_ and CH_3_COCH_3_ was detected. For the O_2_/HAc ratio at 0.28, the hydrogen yield increased to 2.60 mol-H_2_/mol-HAc; with further increasing the O_2_/HAc ratio to 1, the hydrogen yield decreased to 1.37 mol-H_2_/mol-HAc. Simultaneously, CO_2_ selectivity increased, while CO selectivity decreased gradually with more O_2_ in the feeding gas. This indicated that as the O_2_/HAc ratio increased gradually, the water gas shift reaction (CO + H_2_O→CO_2_ + H_2_) could be promoted, leading the reaction to proceed in a more favorable direction. However, if the oxygen concentration was too high, hydrogen was further oxidized into water, reducing the hydrogen yield. To achieve the heat balance and to obtain a high hydrogen yield, the O_2_/HAc ratio at 0.28 can be selected as the optimal feed ratio.

The effect of temperatures was tested under the following conditions: reaction temperature 450–750 °C, GHSV at 51,000 mL·g_catal_^−1^·h^−1^, CH_3_COOH/H_2_O/O_2_ = 1/4/0.28, as shown in Figure 6B. At 450 °C, the acetic acid conversion was only 63.9% with a low hydrogen yield of 0.68 mol-H_2_/mol-HAc and a trace of methane, while the acetone selectivity reached 34.7%, suggesting that the side reaction of ketonization dominated the process at low temperatures [56]. When temperature rose to 550 °C, acetic acid conversion, hydrogen yield and CO_2_/CO selectivity increased gradually, while acetone selectivity decreased significantly. At 700 °C, the hydrogen yield reached 2.60 mol-H_2_/mol-Hac, with selectivity to CO_2_ and CO remaining stable at 57.1% and 40.4%, respectively. As the temperature further increased to 750 °C, the hydrogen yield dropped slightly to 2.39 mol-H_2_/mol-HAc due to the reverse water gas shift reaction. These results demonstrate that 700 °C can be the optimal temperature for ATR of acetic acid.

### 3.4. Characterizations of Spent Catalysts

**Figure 7:**
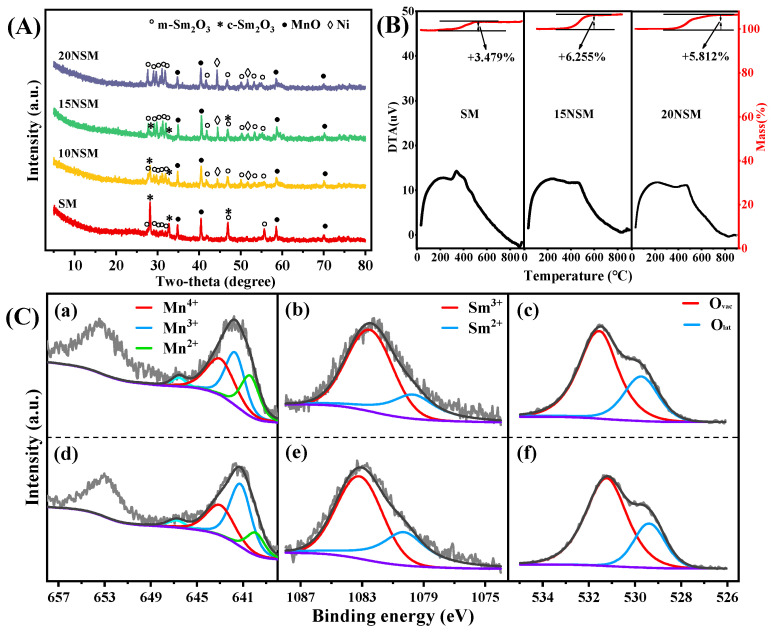
(**A**) XRD patterns and (**B**) TG/DTA profiles of spent xNSM catalysts; (**C**) Mn 2p, Sm 3d and O 1s XPS spectra of spent SM (**a**–**c**) and 15NSM (**d**–**f**) catalysts (dark gray: fit curve; light gray: raw data; purple: background).

The spent catalysts were characterized by XRD to find possible variations after 10 h of ATR at 700 °C, as shown in Figure 7A. For the SM catalyst, peaks of m-Sm_2_O_3_ weakened while peaks of c-Sm_2_O_3_ were enhanced, and similar phenomena existed over the 10NSM catalyst, indicating that phase transition of m-Sm_2_O_3_ to c-Sm_2_O_3_ occurred during the reaction process. Over the 15NSM catalyst, no obvious phase change was observed, suggesting that the Sm_2_O_3_-MnO matrix formed after the reduction offered a stable reaction interface for active Ni^0^ species. Over the 20NSM catalyst, the peaks of m-Sm_2_O_3_ intensified, while the c-Sm_2_O_3_ phase disappeared. In addition, as listed in Table 2, as compared with fresh reduced catalysts, there was no significant increase in the Ni^0^ particle size over these spent catalysts.

The XPS spectra of spent catalysts were recorded as shown in Figure 7C and Table 4. For the SM catalyst, the percentage of Mn^3+^ decreased to 37%, while that of the 15NSM catalyst was recorded at 52%. In addition, the valence variations of Sm were also studied. The Sm^2+^/(Sm^2+^ + Sm^3+^) ratio over the SM catalyst decreased to 29%, while that of 15NSM remained near 36%. The results indicate that after 10 h of ATR reaction, there was still more Mn^3+^ and Sm^2+^ over the surface of 15NSM. Meanwhile, within the spent 15NSM catalyst, the O_vac_/(O_lat_ + O_vac_) ratio was recorded at 73%, which is still higher than that of the SM catalyst at 66%. Furthermore, as shown in Figure 3h, the Ni 2p spectra in the spent 15NSM catalyst indicated that Ni^0^ species remained relatively stable, with the Ni^0^/(Ni^0^ + Ni^2+^) ratio recorded as 35%.

TG/DTA was tested on the spent catalysts to verify carbon deposition, as shown in Figure 7B. For the SM catalyst, a peak of weight gain (3.479%) near 300 °C can be attributed to the oxidation of Mn^2+^ [57]. For the 15NSM catalyst, a significant peak of weight gain (6.255%) appeared at 300–600 °C, which can be attributed to the oxidation of both Mn^2+^ and Ni^0^, suggesting that Ni^0^ still remained within the spent 15NSM catalyst [58]. For the 20NSM catalyst, there was also a peak of weight gain (5.812%), which is lower than that of the 15NSM catalyst and can be attributed to the partial oxidation of Ni^0^ on the catalyst surface. Meanwhile, the weight increasing in the TG curve corresponds exactly to the exothermic peak in DTA. In addition, no weight loss peak of carbon deposits was found in all catalysts because of the interaction between the support Sm_2_O_3_-MnO and metal Ni, as well as surface oxygen vacancies, suggesting that these mullite-derived Ni/Sm_2_O_3_-MnO catalysts exhibited excellent anti-coking ability during the ATR process.

**Figure 8:**
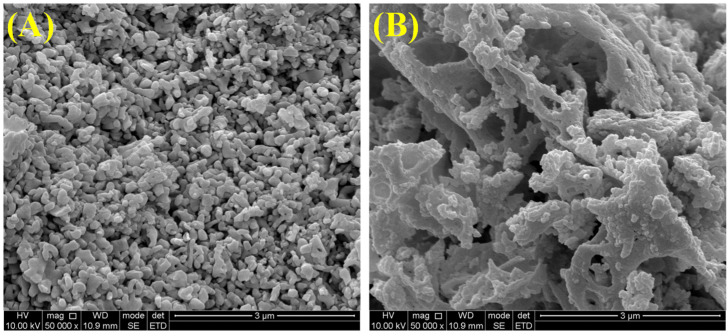
SEM images of spent catalysts: (**A**) SM and (**B**) 15NSM.

The surface morphology and carbon deposition of catalysts were further characterized by SEM images. As shown in Figure 8, the SM catalyst consisted of irregular particles, while coral-like structures were observed on the 15NSM catalyst. Moreover, no obvious carbon deposition was found in these catalysts, which is consistent with the results of TG/DTA.

### 3.5. Discussion

As tested by nitrogen physisorption, the specific surface area of the SM catalyst was 8.0 m^2^/g. After reduction, the mullite-like SmMn_2_O_5_ structure was converted into m-Sm_2_O_3_ and c-Sm_2_O_3_ along with an MnO phase. During the ATR test, the HAc conversion decreased from 75.2% to 55.2%, and only a trace of hydrogen was detected; meanwhile, the selectivity to methane and acetone reached 12.1% and 31.1%, respectively, suggesting that Sm_2_O_3_-MnO mixed oxide cannot effectively convert acetic acid and derived intermediate products (such as CH_3_CO* and CH_3_*) into hydrogen. XRD patterns showed that SM catalyst exhibited an obvious phase transition from m-Sm_2_O_3_ to c-Sm_2_O_3_ after the ATR reaction, indicating that the Sm_2_O_3_-MnO interface was unstable.

For the SmMn_2_O_5_ mullite-derived catalysts loaded with Ni species, with treatment of reduction, the as-formed Sm_2_O_3_-MnO support interacted with the Ni metal and formed an interface of Ni/Sm_2_O_3_-MnO, as shown in XRD and XPS. During the ATR test, the 15NSM catalyst exhibited excellent catalytic performance with a stable HAc conversion of 100% and a higher hydrogen yield of 2.62 mol-H_2_/mol-HAc, while no by-products, such as methane and acetone, were detected. Meanwhile, the 15NSM catalyst presented the highest TOF (0.78 × 10^−2^·s^−1^) and the lowest Ea (29.0 kJ/mol) in kinetic tests, suggesting that the 15NSM catalyst was more active to convert HAc for hydrogen production.

**Scheme 1:**
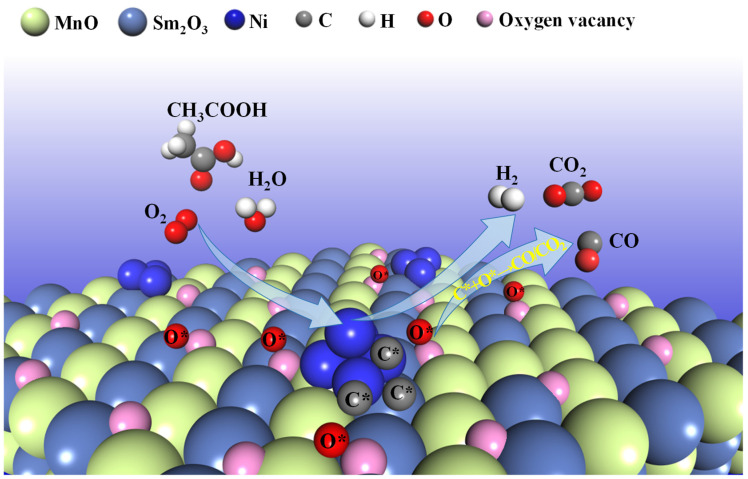
The evolution route of acetic acid over the 15NSM catalyst in ATR of HAc (* represents the intermediates).

The superior catalytic performance of the 15NSM catalyst in the ATR reaction is mainly due to the synergistic interaction between Ni, Mn and Sm species. Firstly, as shown in the XRD, after hydrogen reduction, Sm_2_O_3_-MnO support derived from SmMn_2_O_5_ can interact with the metal Ni to form stable Ni/Sm_2_O_3_-MnO active centers. The electron transfers between Ni and the Sm_2_O_3_-MnO support enhanced the metal–support interaction, which inhibited the tendency of Ni to aggregate, as suggested by the H_2_-TPR results (Figure 2B) and XPS. Because of the interaction between Ni and Sm_2_O_3_-MnO in the 15NSM catalysts, the phase transition from m-Sm_2_O_3_ to c-Sm_2_O_3_ during the ATR reaction was effectively suppressed, which can be proved by XRD, and stability of the catalyst was improved. Meanwhile, over the Ni/Sm_2_O_3_-MnO active centers, the Mn^3+^ species donated electrons to Sm^3+^ and Ni^2+^, and the electron exchange via Mn^3+^ + Sm^3+^ ↔ Mn^4+^ + Sm^2+^ promoted the formation of surface oxygen vacancies in 15NSM, which effectively promoted the migration of active oxygen species O* and accelerated the gasification of carbon precursor C* (C* + O*→CO, CO* + O*→CO_2_) [59,60,61], thus inhibiting coke formation, as shown in TG/DTA, SEM and the proposed Scheme 1. In addition, due to the electron donation effect of the Mn species, a relatively stable surface Ni content was maintained throughout the reaction. Therefore, stable active centers of Ni/Sm_2_O_3_-MnO were obtained in the 15NSM catalyst. The higher TOF and lower Ea values indicated that the 15NSM catalyst exhibited better intrinsic catalytic activity [53], which means that the energy for the Ni/Sm_2_O_3_-MnO active center to activate and transform acetic acid was lower. Therefore, the 15NSM catalyst is more active for acetic acid conversion.

For the 20NSM catalyst, the specific surface area decreased to 7.3 m^2^/g, and the dispersion of the Ni species was only 4.3%, suggesting that excessive Ni species agglomerated and resulted in blockage of catalyst pores. Meanwhile, as shown in the XRD patterns, a larger Ni^0^ particle size of 50.4 nm was found in the 20NSM catalyst. Furthermore, phase transition was also observed over the spent 20NSM catalyst. Therefore, the 20NSM catalyst exhibited poor reactivity in the ATR reaction with HAc conversion, and the hydrogen yield decreased from 100% to 97.1% and 2.52 mol-H_2_/mol-HAc to 2.19 mol-H_2_/mol-HAc, respectively. Moreover, CO and the by-product of CH_4_ gradually increased, indicating that reverse water gas shift and methanation reactions were promoted.

## 4. Conclusions

A series of x%Ni/Sm_2_O_3_-MnO catalysts derived from SmMn_2_O_5_ mullite precursors were prepared by solution combustion and the impregnation method and were tested in auto-thermal reforming of acetic acid. As for the SM catalyst without the Ni species, excellent anti-coke ability was obtained during the ATR reaction; however, due to the lack of an active Ni metal, the SM catalyst produced more by-products, such as methane and acetone, and no target product hydrogen was generated. With Ni species loading on the SmMn_2_O_5_ mullite, the derived 15%Ni/Sm_2_O_3_-MnO (15NSM) catalyst showed superior catalytic performance, with stable HAc conversion near 100% and hydrogen yield at 2.62 mol-H_2_/mol-HAc, as well as a higher TOF of 0.78 × 10^−2^·s^−1^ and lower Ea of 29.0 kJ/mol. It was observed that with appropriate Ni loading on the 15NSM catalyst, the Sm_2_O_3_-MnO support derived from the SmMn_2_O_5_ mullite precursor interacted with the Ni metal, inhibiting phase transition of Sm_2_O_3_ and obtaining stable Ni/Sm_2_O_3_-MnO active centers. More importantly, abundant surface oxygen vacancies were formed by electron transfers (Mn^3+^ + Sm^3+^ ↔ Mn^4+^ + Sm^2+^) in the Sm_2_O_3_-MnO mixed oxides, improving the mobility of oxygen species and promoting the gasification of coke precursors. Meanwhile, the electron donation effect of the Mn species also further constrained the oxidation of Ni^0^ during the reaction. Hence, under an appropriate amount of Ni loading, Ni/Sm_2_O_3_-MnO catalysts can exhibit excellent resistance to coke deposition and oxidation.

## Figures and Tables

**Table 1:** Physicochemical properties and Ni^0^ particles of xNSM catalysts.

Catalysts	Ni^0^ Particle Size Estimated by XRD (nm)		SSA (m^2^/g)	Pore Volume (cm^3^/g)	Average Pore Size (nm)
	Reduced ^a^	Spent ^a^			
SM	-	-	8.0	0.020	2.9
10NSM	31.4	36.5	11.8	0.108	2.7
15NSM	41.6	42.9	15.1	0.048	2.6
20NSM	50.4	53.2	7.3	0.029	2.4

^a^ calculated by Ni^0^ at 44.4° in XRD.

**Table 2:** The surface composition of reduced SM and 15NSM catalysts.

Samples	Mn^3+^/ (Mn^2+^ + Mn^3+^ + Mn^4+^) ^b^	Sm^2+^/(Sm^2+^ + Sm^3+^) ^b^	O_vac_/(O_lat_ + O_vac_) ^b^	Ni^0^/(Ni^0^ + Ni^2+^) ^b^
SM	43%	35%	42%	-
15NSM	55%	40%	64%	32%

^b^ Obtained from XPS.

**Table 3:** Ni^0^ dispersion and TOF values of xNSM catalysts.

Catalysts	Ni^0^ Dispersion (%)	TOF (10^−2^·s^−1^)
10NSM	18.1	0.57
15NSM	11.8	0.78
20NSM	4.3	0.62

**Table 4:** The surface composition of spent SM and 15NSM catalysts.

Samples	Mn^3+^/ (Mn^2+^ + Mn^3+^ + Mn^4+^)	Sm^2+^/(Sm^2+^ +Sm^3+^)	O_vac_/(O_lat_ + O_vac_)	Ni^0^/(Ni^0^ + Ni^2+^)
SM	37%	29%	66%	-
15NSM	52%	36%	73%	31%

## Data Availability

The raw data supporting the conclusions of this article will be made available by the authors on request.

## References

[1] Ren J., Liu Y.-L. (2022). Promoting syngas production from steam reforming of toluene using a highly stable Ni/(Mg, Al)O_x_ catalyst. Appl. Catal. B Environ..

[2] Xiao Z., Li Y., Hou F., Wu C., Pan L., Zou J., Wang L., Zhang X., Liu G., Li G. (2019). Engineering oxygen vacancies and nickel dispersion on CeO_2_ by Pr doping for highly stable ethanol steam reforming. Appl. Catal. B Environ..

[3] Kumar A., Singh R., Sinha A.S.K. (2019). Catalyst modification strategies to enhance the catalyst activity and stability during steam reforming of acetic acid for hydrogen production. Int. J. Hydrogen Energy.

[4] Kapdan I.K., Kargi F. (2006). Bio-hydrogen production from waste materials. Enzym. Microb. Technol..

[5] Palanisamy A., Soundarrajan N., Ramasamy G. (2021). Analysis on production of bioethanol for hydrogen generation. Environ. Sci. Pollut. Res. Int..

[6] Parthasarathy P., Narayanan K.S. (2014). Hydrogen production from steam gasification of biomass: Influence of process parameters on hydrogen yield—A review. Renew. Energy.

[7] Winter C.-J. (2009). Hydrogen energy—Abundant, efficient, clean: A debate over the energy-system-of-change. Int. J. Hydrogen Energy.

[8] Atilhan S., Park S., El-Halwagi M.M., Atilhan M., Moore M., Nielsen R.B. (2021). Green hydrogen as an alternative fuel for the shipping industry. Curr. Opin. Chem. Eng..

[9] Challiwala M.S., Ghouri M.M., Linke P., El-Halwagi M.M., Elbashir N.O. (2017). A combined thermo-kinetic analysis of various methane reforming technologies: Comparison with dry reforming. J. CO_2_ Util..

[10] Santamaria L., Lopez G., Fernandez E., Cortazar M., Arregi A., Olazar M., Bilbao J. (2021). Progress on Catalyst Development for the Steam Reforming of Biomass and Waste Plastics Pyrolysis Volatiles: A Review. Energy Fuels.

[11] Ren J., Cao J.-P., Yang F.-L., Liu Y.-L., Tang W., Zhao X.-Y. (2021). Understandings of Catalyst Deactivation and Regeneration during Biomass Tar Reforming: A Crucial Review. ACS Sustain. Chem. Eng..

[12] Thanigaivel S., Vickram S., Manikandan S., Deena S.R., Subbaiya R., Karmegam N., Govarthanan M., Kim W. (2022). Sustainability and carbon neutralization trends in microalgae bioenergy production from wastewater treatment: A review. Bioresour. Technol..

[13] Zhao B., Hu Y., Gao J., Zhao G., Ray M.B., Xu C.C. (2020). Recent Advances in Hydroliquefaction of Biomass for Bio-oil Production Using In Situ Hydrogen Donors. Ind. Eng. Chem. Res..

[14] Zhang L., Hu X., Hu K., Hu C., Zhang Z., Liu Q., Hu S., Xiang J., Wang Y., Zhang S. (2018). Progress in the reforming of bio-oil derived carboxylic acids for hydrogen generation. J. Power Sources.

[15] Vagia E., Lemonidou A. (2008). Thermodynamic analysis of hydrogen production via autothermal steam reforming of selected components of aqueous bio-oil fraction. Int. J. Hydrogen Energy.

[16] Nabgan W., Tuan Abdullah T.A., Mat R., Nabgan B., Gambo Y., Ibrahim M., Ahmad A., Jalil A.A., Triwahyono S., Saeh I. (2017). Renewable hydrogen production from bio-oil derivative via catalytic steam reforming: An overview. Renew. Sustain. Energy Rev..

[17] Li X., Xue L., Zhu Y., Chen G., Yang G., Wang S. (2018). Mechanistic study of bio-oil catalytic steam reforming for hydrogen production: Acetic acid decomposition. Int. J. Hydrogen Energy.

[18] Chen Y., Zhai Z., Liu J., Zhang J., Geng Z., Lyu H. (2020). Exploring the reaction mechanism of ethanol synthesis from acetic acid over a Ni_2_In(100) surface. Phys. Chem. Chem. Phys..

[19] Jampaiah D., Ippolito S.J., Sabri Y.M., Tardio J., Selvakannan P.R., Nafady A., Reddy B.M., Bhargava S.K. (2016). Ceria–zirconia modified MnO_x_ catalysts for gaseous elemental mercury oxidation and adsorption. Catal. Sci. Technol..

[20] Lin X., Li S., He H., Wu Z., Wu J., Chen L., Ye D., Fu M. (2018). Evolution of oxygen vacancies in MnO_x_-CeO_2_ mixed oxides for soot oxidation. Appl. Catal. B Environ..

[21] Lee G., Kim D., Kwak B.S., Kang M. (2014). Hydrogen rich production by ethanol steam reforming reaction over Mn/Co_10_Si_90_MCM-48 catalysts. Catal. Today.

[22] Wang Y., Li L., Cui C., Da Costa P., Hu C. (2022). The effect of adsorbed oxygen species on carbon-resistance of Ni-Zr catalyst modified by Al and Mn for dry reforming of methane. Catal. Today.

[23] An S., Zhang Y., Hu X., Xie X., Wang Q., Chen H., Huang L. (2020). Durable Mn(II)Cr(III)O_x_ composites-supported Ni-based catalysts with wide dynamic range for hydrogen production via auto-thermal reforming of acetic acid. Fuel.

[24] Song Y., Chen B., Hu X., Wang Q., Xie X., Dai H., Huang L. (2020). Highly Efficient Al-Doped Ni-Mn-O Catalysts for Auto-Thermal Reforming of Acetic Acid: Role of MnAl_2_O_4_ for Stability of Ni Species. Energy Fuels.

[25] Mo S., Zhang Q., Li J., Sun Y., Ren Q., Zou S., Zhang Q., Lu J., Fu M., Mo D. (2020). Highly efficient mesoporous MnO_2_ catalysts for the total toluene oxidation: Oxygen-Vacancy defect engineering and involved intermediates using in situ DRIFTS. Appl. Catal. B Environ..

[26] Deng H., Kang S., Ma J., Wang L., Zhang C., He H. (2019). Role of Structural Defects in MnO_x_ Promoted by Ag Doping in the Catalytic Combustion of Volatile Organic Compounds and Ambient Decomposition of O_3_. Environ. Sci. Technol..

[27] Shi C., Zhang P. (2012). Effect of a second metal (Y, K, Ca, Mn or Cu) addition on the carbon dioxide reforming of methane over nanostructured palladium catalysts. Appl. Catal. B Environ..

[28] Zhang W.D., Liu B.S., Tian Y.L. (2007). CO_2_ reforming of methane over Ni/Sm_2_O_3_-CaO catalyst prepared by a sol–gel technique. Catal. Commun..

[29] Duarte R.B., Nachtegaal M., Bueno J.M.C., van Bokhoven J.A. (2012). Understanding the effect of Sm_2_O_3_ and CeO_2_ promoters on the structure and activity of Rh/Al_2_O_3_ catalysts in methane steam reforming. J. Catal..

[30] Zheng Y., Thampy S., Ashburn N., Dillon S., Wang L., Jangjou Y., Tan K., Kong F., Nie Y., Kim M.J. (2019). Stable and Active Oxidation Catalysis by Cooperative Lattice Oxygen Redox on SmMn_2_O_5_ Mullite Surface. J. Am. Chem. Soc..

[31] Li Z., Hu X., Zhang L., Liu S., Lu G. (2012). Steam reforming of acetic acid over Ni/ZrO_2_ catalysts: Effects of nickel loading and particle size on product distribution and coke formation. Appl. Catal. A Gen..

[32] Chen Q., Liao F., Ding C., Hu X., Xu Y., Cheng P., Zheng Z., Huang L., Wang N. (2024). Reaction induced Ni/MgTi_2_O_5_ interface promotes the resistance to sintering and oxidation in auto-thermal reforming of acetic acid. Catal. Today.

[33] Cychosz K.A., Guillet-Nicolas R., Garcia-Martinez J., Thommes M. (2017). Recent advances in the textural characterization of hierarchically structured nanoporous materials. Chem. Soc. Rev..

[34] Xu L., Zhang J., Ding J., Liu T., Shi G., Li X., Dang W., Cheng Y., Guo R. (2020). Pore Structure and Fractal Characteristics of Different Shale Lithofacies in the Dalong Formation in the Western Area of the Lower Yangtze Platform. Minerals.

[35] Tavanarad M., Meshkani F., Rezaei M. (2018). Production of syngas via glycerol dry reforming on Ni catalysts supported on mesoporous nanocrystalline Al_2_O_3_. J. CO_2_ Util..

[36] Lang Y., Zhang J., Feng Z., Liu X., Zhu Y., Zeng T., Zhao Y., Chen R., Shan B. (2018). CO oxidation over MO_x_ (M = Mn, Fe, Co, Ni, Cu) supported on SmMn_2_O_5_ composite catalysts. Catal. Sci. Technol..

[37] Liu H., Hadjltaief H.B., Benzina M., Gálvez M.E., Da Costa P. (2019). Natural clay based nickel catalysts for dry reforming of methane: On the effect of support promotion (La, Al, Mn). Int. J. Hydrogen Energy.

[38] Liu Q., Yang H., Dong H., Zhang W., Bian B., He Q., Yang J., Meng X., Tian Z., Zhao G. (2018). Effects of preparation method and Sm_2_O_3_ promoter on CO methanation by a mesoporous NiO-Sm_2_O_3_/Al_2_O_3_ catalyst. New J. Chem..

[39] Ayub N.A., Bahruji H., Mahadi A.H. (2021). Barium promoted Ni/Sm_2_O_3_ catalysts for enhanced CO_2_ methanation. RSC Adv..

[40] Si W., Wang Y., Peng Y., Li J. (2015). Selective Dissolution of A-Site Cations in ABO_3_ Perovskites: A New Path to High-Performance Catalysts. Angew. Chem. Int. Ed..

[41] Liu G., Liu J., Li W., Liu C., Wang F., He J., Guild C., Jin J., Kriz D., Miao R. (2017). Aerobic oxidation of alcohols over Ru-Mn-Ce and Ru-Co-Ce catalysts: The effect of calcination temperature. Appl. Catal. A Gen..

[42] Biesinger M.C., Payne B.P., Grosvenor A.P., Lau L.W.M., Gerson A.R., Smart R.S.C. (2011). Resolving surface chemical states in XPS analysis of first row transition metals, oxides and hydroxides: Cr, Mn, Fe, Co and Ni. Appl. Surf. Sci..

[43] Ilton E.S., Post J.E., Heaney P.J., Ling F.T., Kerisit S.N. (2016). XPS determination of Mn oxidation states in Mn (hydr)oxides. Appl. Surf. Sci..

[44] Sun C., Liu H., Chen W., Chen D., Yu S., Liu A., Dong L., Feng S. (2018). Insights into the Sm/Zr co-doping effects on N_2_ selectivity and SO_2_ resistance of a MnO_x_-TiO_2_ catalyst for the NH_3_-SCR reaction. Chem. Eng. J..

[45] Li Y., Wang H., Zhang R., Yang R. (2019). Double redox couples manganese oxide nanorods with tunable oxygen defects and their catalytic combustion properties. J. Nanopart. Res..

[46] Ma C., Wen Y., Rong C., Zhang N., Zheng J., Chen B.H. (2017). δ-MnO_2_ with an ultrahigh Mn^4+^ fraction is highly active and stable for catalytic wet air oxidation of phenol under mild conditions. Catal. Sci. Technol..

[47] Liu L., Zhang H., Guo M., Zhou P., Min X., Jia J., Sun T. (2019). Self-molten-polymerization synthesis of highly defected Mn/Sm binary oxides with mesoporous structures for efficient removal of toluene and chlorobenzene. Inorg. Chem. Front..

[48] Yu Y., Ji J., Li K., Huang H., Shrestha R.P., Kim Oanh N.T., Winijkul E., Deng J. (2020). Activated carbon supported MnO nanoparticles for efficient ozone decomposition at room temperature. Catal. Today.

[49] Wang N., Qian W., Chu W., Wei F. (2016). Crystal-plane effect of nanoscale CeO_2_ on the catalytic performance of Ni/CeO_2_ catalysts for methane dry reforming. Catal. Sci. Technol..

[50] Liu B., Li C., Zhang G., Yao X., Chuang S.S.C., Li Z. (2018). Oxygen Vacancy Promoting Dimethyl Carbonate Synthesis from CO_2_ and Methanol over Zr-Doped CeO_2_ Nanorods. ACS Catal..

[51] Do J.Y., Park N.-K., Seo M.W., Lee D., Ryu H.-J., Kang M. (2020). Effective thermocatalytic carbon dioxide methanation on Ca-inserted NiTiO_3_ perovskite. Fuel.

[52] Adhikari S., Fernando S., Gwaltney S., Filipto S., Markbricka R., Steele P., Haryanto A. (2007). A thermodynamic analysis of hydrogen production by steam reforming of glycerol. Int. J. Hydrogen Energy.

[53] Fedorova V., Simonov M., Valeev K., Bespalko Y., Smal E., Eremeev N., Sadovskaya E., Krieger T., Ishchenko A., Sadykov V. (2021). Kinetic Regularities of Methane Dry Reforming Reaction on Nickel-Containing Modified Ceria-Zirconia. Energies.

[54] Escobar J., Reyes J.A.D.L., Viveros T., Barrera M.C. (2006). Cyclohexane Dehydrogenation over Wet-Impregnated Ni on Al_2_O_3_-TiO_2_ Sol-Gel Oxides. Ind. Eng. Chem. Res..

[55] Nakhaei Pour A., Mousavi M. (2015). Combined reforming of methane by carbon dioxide and water: Particle size effect of Ni-Mg nanoparticles. Int. J. Hydrogen Energy.

[56] Imada S., Peng X., Cai Z., Najib A., Miyauchi M., Abe H., Fujita T. (2020). NiYAl-Derived Nanoporous Catalysts for Dry Reforming of Methane. Materials.

[57] Wang F., Liu B.S., Zhang Z.F., Zheng S. (2015). High-Temperature Desulfurization of Coal Gas over Sm Doped Mn-based/MSU-S Sorbents. Ind. Eng. Chem. Res..

[58] Nabgan W., Abdullah T.A.T., Mat R., Nabgan B., Jalil A.A., Firmansyah L., Triwahyono S. (2017). Production of hydrogen via steam reforming of acetic acid over Ni and Co supported on La_2_O_3_ catalyst. Int. J. Hydrogen Energy.

[59] Xie Q., Zhang H., Kang J., Cheng J., Zhang Q., Wang Y. (2018). Oxidative Dehydrogenation of Propane to Propylene in the Presence of HCl Catalyzed by CeO_2_ and NiO-Modified CeO_2_ Nanocrystals. ACS Catal..

[60] Ding C., Hu X., Sun W., Hailili R., Liao F., Shu C., Huang J., Huang L., Wang N. (2023). Interface of Ni-MgCr_2_O_4_ Spinel Promotes the Autothermal Reforming of Acetic Acid through Accelerated Oxidation of Carbon-Containing Intermediate Species. ACS Catal..

[61] Hu X., Yang J., Sun W., Wang N., An S., Wang Q., Zhang Y., Xie X., Huang L. (2020). Y-Zr-O solid solution supported Ni-based catalysts for hydrogen production via auto-thermal reforming of acetic acid. Appl. Catal. B Environ..

